# Bufotalin Induces Oxidative Stress-Mediated Apoptosis by Blocking the ITGB4/FAK/ERK Pathway in Glioblastoma

**DOI:** 10.3390/antiox13101179

**Published:** 2024-09-27

**Authors:** Junchao Tan, Guoqiang Lin, Rui Zhang, Yuting Wen, Chunying Luo, Ran Wang, Feiyun Wang, Shoujiao Peng, Jiange Zhang

**Affiliations:** Innovation Research Institute of Traditional Chinese Medicine, Shanghai University of Traditional Chinese Medicine, Shanghai 201203, China; tanjunchao@shutcm.edu.cn (J.T.); lingq@sioc.ac.cn (G.L.); rui_zhang@shutcm.edu.cn (R.Z.); wenyuting@shutcm.edu.cn (Y.W.); 0012021193@shutcm.edu.cn (C.L.); 12022215@shutcm.edu.cn (R.W.)

**Keywords:** bufotalin, apoptosis, oxidative stress, ITGB4, glioblastoma

## Abstract

Bufotalin (BT), a major active constituent of Chansu, has been found to possess multiple pharmacological activities. Although previous studies have shown that BT could inhibit the growth of glioblastoma (GBM), the safety of BT in vivo and the potential mechanism are still unclear. We conducted a systematic assessment to investigate the impact of BT on GBM cell viability, migration, invasion, and colony formation. Furthermore, in vivo results were obtained to evaluate the effect of BT on tumor growth. The preliminary findings of our study demonstrate the effective inhibition of GBM cell growth and subcutaneous tumor development in mice by BT, with tolerable levels of tolerance observed. Mechanistically, BT treatment induced mitochondrial dysfunction, bursts of reactive oxygen species (ROS), and subsequent cell apoptosis. More importantly, proteomic-based differentially expressed proteins analysis revealed a significant downregulation of integrin β4 (ITGB4) following BT treatment. Furthermore, our evidence suggested that the ITGB4/focal adhesion kinase (FAK)/extracellular signal-related kinase (ERK) pathway involved BT-induced apoptosis. Overall, our study demonstrates the anti-GBM effects of BT and elucidates the underlying mechanism, highlighting BT as a potential therapeutic option for GBM.

## 1. Introduction

Originating from the neural ectoderm, gliomas are the most commonly diagnosed primary malignant tumors of the CNS [1], and they exhibit diffuse growth, high invasiveness, and generally poor prognosis [2,3]. Gliomas are graded on a scale of 1 to 4 and account for almost 30% of all brain tumors [4]. Glioblastoma (GBM), classified as grade 4, is the deadliest subtype, with a median overall survival of only 14.4 months [5]. Radiotherapy and chemotherapy with the alkylating agent temozolomide (TMZ) after surgical resection is the mainstay of treatment for GBM. It is known that TMZ is administrated to disrupt the DNA of tumor cells and result in cell death [6]. However, the O6-methylguanine-DNA methyltransferase (MGMT) gene, which encodes a DNA repair enzyme, and its overexpression diminishes the effects of chemotherapy and induces TMZ resistance [7]. Therefore, it is imperative to investigate more effective therapy approaches and targets for GBM.

**Figure 1:**
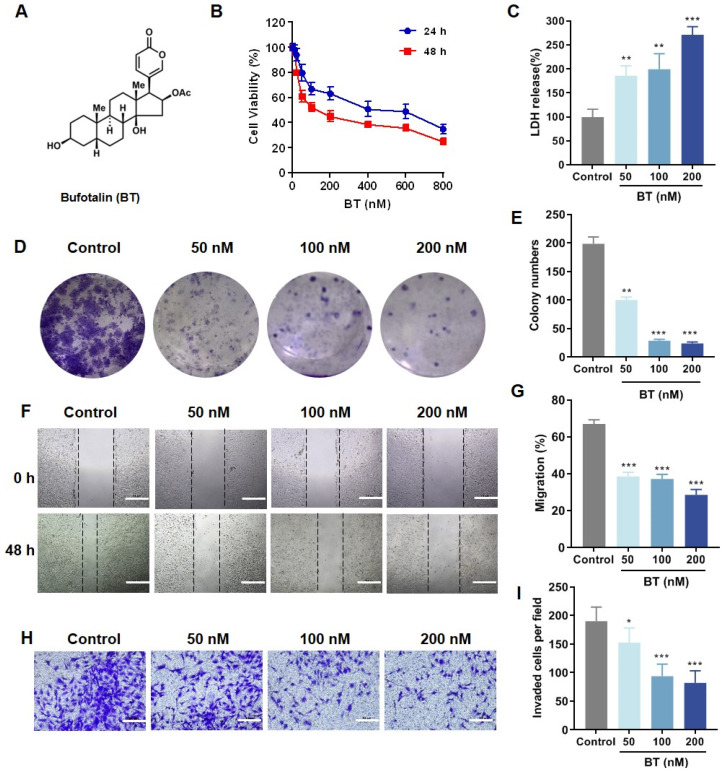
BT inhibits the growth of U251 cells. (**A**) Chemical structure of BT. (**B**) Cell viability of U251 cells treated with BT for 24 h and 48 h. (**C**) Cytotoxicity was evaluated by LDH release assay. (**D**,**E**) Colony formation and quantitative measurements of colony formation in U251 cells treated with BT. (**F**,**G**) Cell migration and statistical analysis of U251 cells treated with BT (scale bar: 500 μm). (**H**,**I**) Invasion ability and statistical analysis of U251 cells were detected by transwell assay (scale bar: 500 μm). All data were expressed as the mean ± SD of three independent experiments. * *p* < 0.05, ** *p* < 0.01, *** *p* < 0.001 compared with control.

Traditional Chinese medicine (TCM) has gained increasing attention in cancer therapy because of its low toxicity and minimal side effects [8,9]. Chansu, a TCM extracted from the dried secretions of *Bufo gargarizans Cantor* and *Bufo melanostictus Schneider*, is widely used to treat various conditions, including inflammation, pain, cardiac illness, and cancer [10]. The major components extracted and identified from Chansu were bufadienolides, indole alkaloids, and sterols [11]. Bufotalin (BT) is a bioactive substance among bufadienolides, and its specific chemical structure is shown in Figure 1A. BT exerts anti-tumor effects in various cancer cells, such as osteoblastoma [12], breast cancer [13], esophageal squamous cell carcinoma [14], hepatocellular carcinoma [15], non-small cell lung cancer [16], and melanoma [17]. In addition, previous investigations revealed that BT could enter the brain by overcoming the blood-brain barrier [18]. Although a recent study reported that BT exhibited an anti-tumor effect in GBM cells by inhibiting epithelial-mesenchymal transition, indicating that BT is a potential natural product for combating malignant brain gliomas [19], the anti-glioma effect in vivo and the potential mechanisms of BT still need to be systematically explored.

As transmembrane receptors, integrins mediate interactions with the extracellular matrix (ECM) and regulate many cellular behaviors, including proliferation, migration, and survival [20,21]. Among these receptors, integrin β4 (ITGB4), which is highly expressed in human glioma tissues [22], mainly interacts with a key element of the ECM, namely laminin. Focal adhesion kinase (FAK) is a key downstream molecule of ITGB4. It is recruited and bound to the β subunit of integrin and regulates integrin signaling by phosphorylation at tyrosine 397 [23]. This phosphorylation process subsequently triggers the activation of the downstream PI3K/AKT or ERK signaling pathways [24]. It is known that the ERK signaling pathway plays a pivotal role in various fundamental cellular processes, such as proliferation, survival, differentiation, and migration [25]. Several studies have reported that the metastasis of breast cancer [26] or esophageal squamous cell carcinoma [27] is enhanced via the activation of the ITGB4/FAK/ERK signaling pathway. Nonetheless, the role of ITGB4 in GBM remains unclear.

As the vital organelles in cells, mitochondria serve as the target core for multiple cellular functions, including cellular metabolism, ionic homeostasis, production of reactive oxygen species (ROS), and initiation of apoptosis [28]. Recent studies revealed that mitochondrial dysfunction induced by mitochondrial DNA mutation [29], Ca^2+^ overload [30], or mitochondrial permeability transition [31] has become an effective strategy in cancer therapy [32]. Moreover, as a byproduct of the mitochondrial electron transport chain, ROS production is often highly associated with mitochondrial dysfunction and eventually leads to oxidative stress, which promotes cancer cell death [33]. In the present study, we observed that BT displayed overt anticancer activity against GBM cells both in vitro and in vivo. In addition, utilizing the quantitative proteomics method, we found that ITGB4 was significantly down-regulated. Furthermore, the ITGB4/FAK/ERK pathway was blocked, eventually leading to mitochondrial dysfunction and oxidative stress in U251 cells. In summary, our study underscores the promising therapeutic potential of BT in treating GBM.

## 2. Materials and Methods

### 2.1. Cell Culture

Human GBM cell lines (U251 and U87 MG) were obtained from the Cell Bank of the Chinese Academy of Sciences (Shanghai, China). Cells were maintained in Dulbecco’s modified Eagle’s medium (DMEM; Hakata, Shanghai, China), supplemented with 10% fetal bovine serum (FBS; Serana, Aidenbach, Germany) and 1% penicillin-streptomycin (Hakata) at 37 °C in a humidified atmosphere containing 5% CO_2_.

### 2.2. Cell Viability and Cytotoxicity Assays

U251 cells (5 × 10^3^/well) were seeded in 96-well microplates and a range of BT (Caoyuankang, Chengdu, China) concentrations were added when the cells reached 80% confluence. After 48 h of treatment, cell viability was assessed via a cell counting kit-8 (CCK-8; Topscience, Shanghai, China), and cytotoxicity was measured via a lactate dehydrogenase (LDH) kit (Beyotime, Shanghai, China).

### 2.3. Cell Migration and Invasion Assays

U251 cells (1 × 10^6^/well) were seeded in 6-well plates. Then, wounds were made in each well using 200 μL tips until confluent monolayers were formed. Following this, the cells were incubated in DMEM containing 0.1% FBS and different concentrations of BT for 48 h. Images were captured at 0 h and 48 h following scratch using an inverted microscope (Nikon, Tokyo, Japan).

To detect cell invasion ability, Transwell chambers (8 μm) (Corning, Kennebunk, ME, USA) pre-coated with Matrigel (Chuanqiu, Shanghai, China) were used. In the procedure, U251 cells (4 × 10^4^) were resuspended with FBS-free DMEM into the upper chamber. After incubation with or without BT for 48 h, cells in the lower chamber were subjected to fixation with 4% paraformaldehyde (PFA; Adamas, Shanghai, China) for 30 min, followed by staining with crystal violet (Beyotime, Shanghai, China) for 20 min. Finally, cells were counted in three random fields under a microscope (Nikon, Tokyo, Japan).

### 2.4. Colony Formation Assay

After U251 cells (2 × 10^3^ cells/well) were seeded and treated with BT, they were maintained in a cell incubator for 14 days. Finally, the colonies were stained with crystal violet for 15 min and photographed.

### 2.5. Tumor Xenograft Experiments

Male BALB/c-nu mice (18–20 g) were purchased from SLAC (Shanghai, China) and housed under standard conditions. U251 cells were subcutaneously injected into the right flanks of mice. Subsequently, mice harboring U251 xenografts were divided into five groups and intraperitoneally administered control (saline containing 0.1% DMSO), 1 mg/kg BT, 2 mg/kg BT, 5 mg/kg BT, or TMZ (20 mg/kg) daily. Tumor volume (mm^3^) was calculated as (length × width^2^)/2. After 14 days, the mice were euthanized, and xenograft tumors, blood, and the main organs were collected. All animal experiments were approved by the Ethics Committee of the Shanghai University of Traditional Chinese Medicine.

### 2.6. Cell Death Manner Assay

U251 cells (5 × 10^3^/well) were seeded in 96-well microplates and treated with or without 200 nM BT. Further, 10 μM Z-VAD-FMK (Topscience), 10 μM necrostatin-1 (Topscience), and 10 μM ferrostatin-1 (Yeasen, Shanghai, China) were co-incubated with BT. After 48 h, 10 μL CCK-8 was added for an extra 1 h. Finally, the absorbance was measured at 450 nm.

### 2.7. Apoptosis Assay

Apoptosis was first assessed by Hoechst 33258 staining according to previously reported specifications [34]. Following a 48 h incubation period with or without BT, the U251 cells were fixed with 4% PFA and stained with Hoechst 33258 (Beyotime) in the dark for 30 min before imaging them under an inverted fluorescence microscope.

Apoptotic cells were determined using an Annexin V-FITC/propidium iodide (PI) Apoptosis Detection Kit (Meilun, Dalian, China). Briefly, U251 cells were harvested and resuspended in 500 μL binding buffer supplemented with 5 μL of AnnexinV-FITC and 5 μL of PI. After incubation for 5 min, cells were then analyzed by flow cytometry (Beckman Coulter, Suzhou, China).

### 2.8. Quantitative Proteomic Analysis

Tandem mass tag (TMT) labeling quantitative proteomic analysis was used to identify the differential expression of proteins (DEPs) in U251 cells treated with BT. This procedure was entrusted to Novogene (Beijing, China). Briefly, cells were collected and lysed with radioimmunoprecipitation assay buffer, and the protein samples were digested with trypsin into peptides, followed by the addition of a TMT labeling reagent. Finally, the proteins were analyzed and identified by liquid chromatography-tandem mass spectrometry.

### 2.9. Bioinformatic Analysis

DEPs were visualized using volcano plot analyses (https://www.bioinformatics.com.cn/ (accessed on 23 January 2024)), and the pathway enrichment analysis was conducted using the DAVID database (https://david.ncifcrf.gov/ (accessed on 29 February 2024)). Protein-protein interactions (PPI) were performed using the STRING database (https://string-db.org/ (accessed on 29 February 2024)).

### 2.10. Detection of Adenosine Triphosphate (ATP) and Intracellular Calcium Concentration

After U251 cells (2 × 10^4^ cells/well) were seeded and treated with BT, the ATP level was assessed using an ATP assay kit (Beyotime). For the detection of intracellular calcium, cells were washed with phosphate-buffered saline (PBS) and stained with 200 μL Fluo-4 AM (Beyotime) for 30 min, subsequently photographed and quantified with a fluorescence microscope (Nikon, Tokyo, Japan).

### 2.11. Measurement of Mitochondrial Membrane Potential (MMP) and Mitochondrial Permeability Transition Pore (mPTP)

U251 cells (2 × 10^4^/dish) were cultured in confocal laser dishes and treated with BT for 48 h. Afterward, cells were stained with JC-1 (Beyotime) for 20 min. After that, cells were imaged using a confocal microscope (Nikon, Tokyo, Japan).

The mPTP opening was detected using an mPTP kit (Beyotime). Briefly, the cells were stained with calcein AM and fluorescence quenching solutions for 30 min. Next, the solution was replaced by preheated DMEM and incubated for another 30 min. Finally, cells were imaged using a fluorescence microscope after washing with PBS.

### 2.12. Immunofluorescence and IHC Staining

U251 cells (2 × 10^4^ cells/well) were seeded and treated with BT for 48 h. Then, the cells were fixed with 4% PFA and permeabilized with 0.2% Triton X-100. After staining with γ-H2AX (Beyotime, #C2035S) or Bim (Beyotime, #AF1573) overnight, the cells were incubated with AF488-conjugated secondary antibody (Beyotime, Shanghai, China) for 1 h. Finally, the nuclei were stained with 4, 6-diamidino-2-phenylindole (DAPI) for 5 min before images were taken with an inverted fluorescence microscope.

IHC was performed based on previously established guidelines [35]. We deparaffinized and rehydrated the tumor or main organ slides with xylene and ethanol (Sinopharm chemical reagent, Shanghai, China). After antigen retrieval with 10 mM sodium citrate buffer (Servicebio, Wuhan, China) and blocking with serum for 30 min, the slides were incubated with primary antibodies against Ki-67 (1:200, Cell Signaling Technology, Danvers, MA, USA, #9027T), Bcl-2 (1:200, Cell Signaling Technology, #3498T), and Bax (1:200, Cell Signaling Technology, #5023T). Then, the slides were incubated with horseradish peroxidase (HRP)-labeled secondary antibodies (1:1000, Beyotime) for 50 min, followed by incubation with diaminobenzidine (DAB; Servicebio) and counterstaining with hematoxylin (Servicebio).

### 2.13. Detection of the Levels of ROS, Total Thiol, SOD, GPx and MDA

For ROS detection, after being seeded (2 × 10^4^ cells/well) and treated with BT for 48 h, the U251 cells were stained with 5 μM 2′,7′-dichlorofluorescein diacetate (DCFH-DA) (Beyotime) for 20 min. Subsequently, the cells were imaged using an inverted fluorescence microscope. For detection of mitochondrial superoxide production, the cells were incubated with 5 μM MitoSox Red (Beyotime) for 30 min. Then, the cells were collected and analyzed with a flow cytometer. For total thiol content detection, the cells were collected and mixed with 5 mM DTNB (pH 8.0) for 5 min, and the optical absorbance was measured at 412 nm. For SOD detection, SOD enzyme activity was determined using a SOD assay kit (Jiancheng, Nanjing, China). For GPx detection, the total Glutathione Peroxidase Assay Kit (Beyotime) was used according to the manufacturer’s instructions. For MDA analysis, 200 μL thiobarbituric acid solution (Beyotime) was added to 100 μL cell lysate and incubated at 100 °C for 15 min. Then, a 200 μL mixture was placed on a 96-well microplate, and the absorbance was measured at 532 nm.

### 2.14. Western Blot

Cells were lysed with RIPA buffer, and the protein concentrations were determined using a bicinchoninic acid kit (Meilun). Proteins were separated on 6–12% SDS-PAGE; then transferred onto polyvinylidene difluoride membranes. After blocking with non-fat milk (5%), the membranes were incubated overnight at 4 °C with primary antibodies against ITGB4 (Beyotime, #AF7311), FAK (Beyotime, #AF1108), Phospho-FAK (Tyr397; Beyotime, #AF1960), ERK1/2 (Cell Signaling Technology, #4695T), Phospho-ERK1/2 (Thr202/Tyr204; Cell Signaling Technology, #4377T), Bcl-2, Bax, Bim, cytochrome c (Cell Signaling Technology, #11940), cleaved caspase-9 (Cell Signaling Technology, #7237T), cleaved caspase-3 (Cell Signaling Technology, #9664T), and cleaved PARP (Beyotime, #AF1567). Afterwards, the membranes were incubated with secondary antibodies at room temperature for 2 h. Finally, the protein bands were detected using chemiluminescence reagents (Meilun), and images were quantified using Image J software (version 1.53k).

### 2.15. Statistical Analysis

Statistical analyses were performed using GraphPad Prism version 9. Data were presented as the mean ± standard deviation (SD), and statistical significance was evaluated using Student’s *t*-test or ANOVA followed by Duncan’s test. *p* < 0.05 was considered statistically significant.

## 3. Results

### 3.1. BT Alleviates the Malignancy of GBM Cells In Vitro

To investigate the anti-tumor effects of BT, we treated human GBM cells (U251 and U87 MG) with various concentrations of BT for 48 h. CCK-8 assays demonstrated that BT preferentially killed U251 cells (IC_50_ = 0.15 μM) rather than U87 MG cells (IC_50_ = 0.58 μM). We then found that BT suppressed cell viabilities in a dose- and time-dependent manner (Figure 1B) and significantly induced U251 cell death, as evidenced by increased LDH release (Figure 1C). Colony formation assay also indicated that BT inhibited U251 cell growth (Figure 1D,E). Furthermore, BT significantly inhibited cell migration (Figure 1F,G) and invasion in a dose-dependent manner (Figure 1H,I). Collectively, these data illustrated that BT exhibited anti-tumor effects on U251 cells.

### 3.2. BT Suppresses GBM Cell Growth In Vivo

**Figure 2:**
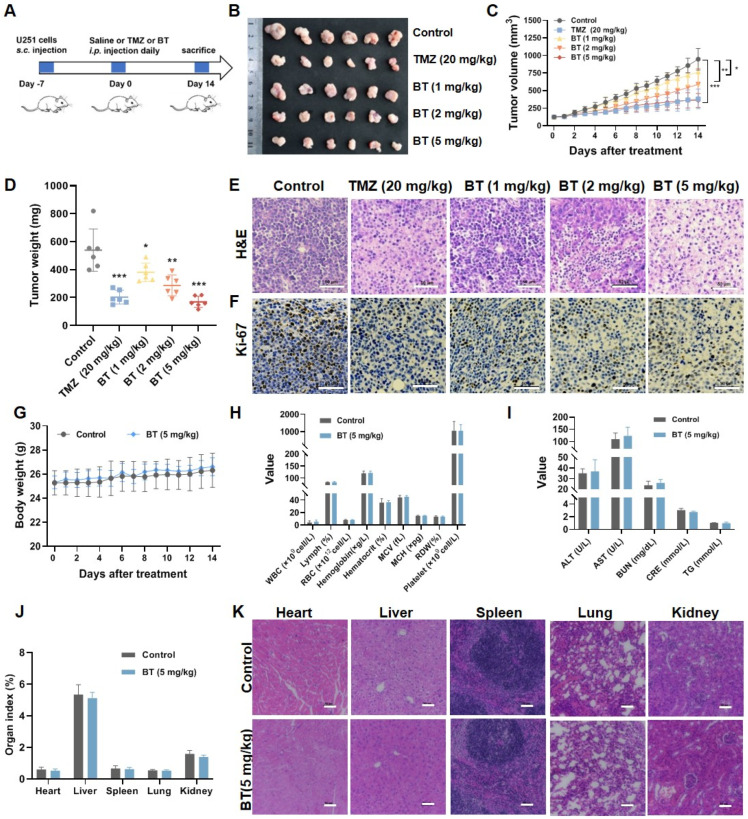
In vivo anti-glioma activity and safety of BT in vivo. (**A**) Schematic illustration of establishing a mouse xenograft model treated with U251 in vivo. (**B**) Representative images of tumors in different groups on day 14. (**C**) Tumor volume was monitored daily during treatment. (**D**) Tumor weight after treatment on day 14. (**E**,**F**) Representative images of H&E and Ki-67 staining of tumor tissues after treatment on day 14 (scale bar: 50 μm). (**G**) Changes in body weight were monitored daily during treatment. (**H**) Hematological analysis of mice treated with BT (5 mg/kg) or saline for 14 days. (**I**) Biochemical analysis of mice treated with BT (5 mg/kg) or saline for 14 days. (**J**) Effects of BT on organ indices in mice treated with BT (5 mg/kg) or saline for 14 days. (**K**) Representative images of H&E-stained mouse organs, including the heart, liver, spleen, lungs, and kidneys (scale bar: 50 μm). All data were expressed as mean ± SD (*n* = 6). * *p* < 0.05, ** *p* < 0.01, *** *p* < 0.001 compared with control.

To study the anti-tumor activity of BT on GBM in vivo, nude mice were subcutaneously injected with U251 cells and grouped when the tumors reached ~100 mm^3^. Then, the mice were treated with BT (1, 2, and 5 mg/kg), TMZ (20 mg/kg), or saline daily for 2 weeks (Figure 2A). As shown in Figure 2B–D, BT treatment induced a marked dose-dependent decrease in tumor volumes and weights. H&E staining revealed that tumors from BT-treated mice (1, 2, and 5 mg/kg) contained fewer cells but increased gaps compared to the control group (Figure 2E). Furthermore, IHC testing showed that BT treatment suppressed Ki-67 (a marker of proliferation) levels in tumor xenograft mice (Figure 2F). Notably, the safety of BT administration in vivo was determined. Compared to the control groups, there were no significant differences in body weight (Figure 2G), and hematological and blood biochemical parameters such as white blood cell (WBC), red blood cell (RBC), mean corpuscular volume (MCV), mean corpuscular hemoglobin (MCH), red blood cell distribution width (RDW), alanine transaminase (ALT), aspartate transaminase (AST), urea nitrogen (BUN), triglyceride (TG) and creatinine (CRE) (Figure 2H,I). These results demonstrated that BT had no significant toxicity to the liver and kidney. In addition, organ index (Figure 2J) and H&E staining (Figure 2K) showed no significant pathological changes in the heart, liver, spleen, lung, and kidney. Our results revealed that BT inhibited GBM growth and was well-tolerated without significant side effects.

### 3.3. BT Induces Apoptosis of U251 Cells

**Figure 3:**
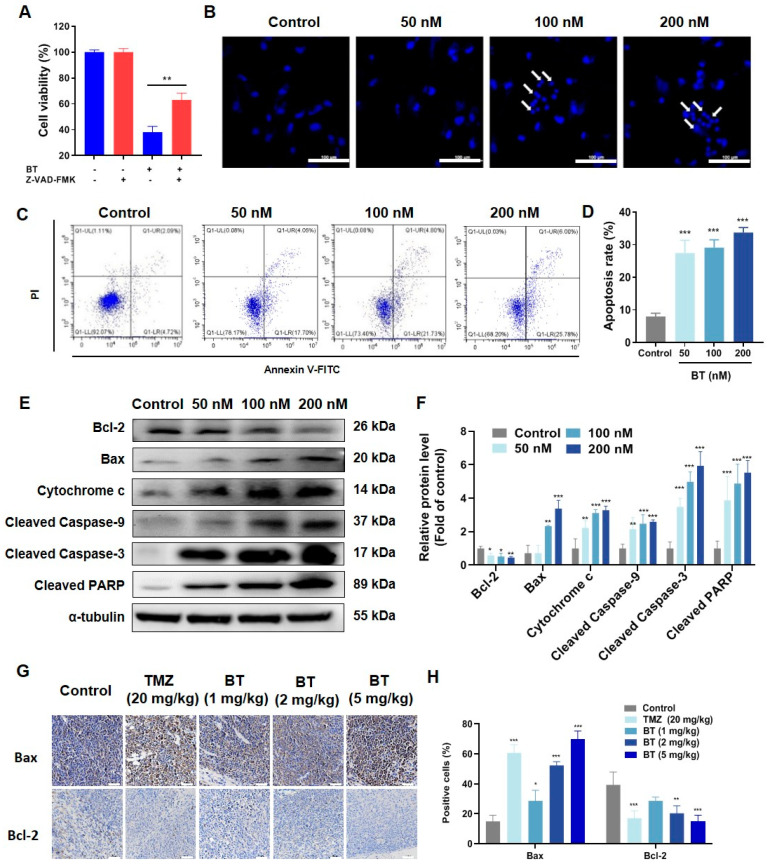
BT-induced apoptosis in U251 cells. (**A**) Cell viability after treatment with a combination of BT (200 nM) and the apoptosis inhibitor Z-VAD-FMK (10 μM) for 48 h. (**B**) Representative images of U251 cell apoptosis after Hoechst 33258 staining in each group (scale bar: 100 μm). Arrows indicate apoptotic cells. (**C**) Apoptosis assay of BT-treated U251 cells. (**D**) The apoptosis rate in U251 cells treated with BT. (**E**) Western blot analysis of apoptosis-related proteins in U251 cells treated with different concentrations of BT for 48 h. (**F**) Statistical analysis of western blots. (**G**) Representative images of Bax and Bcl-2 staining of tumor tissues after treatment on day 14 (scale bar: 50 μm). (**H**) The relative expressions of Bax and Bcl-2 in (**G**). All data were expressed as the mean ± SD of three independent experiments. * *p* < 0.05, ** *p* < 0.01, *** *p* < 0.001 compared with control.

To ascertain the mechanisms underlying BT-induced cell death, we co-incubated U251 cells with BT and inhibitors of ferroptosis (ferrostatin-1), necrosis (necrostatin-1), and apoptosis (Z-VAD-FMK). The results indicated that ferrostatin-1 and necrostatin-1 did not affect the inhibitory effects of BT on cell viability (Figure S1A,B). In contrast, Z-VAD-FMK significantly reduced the efficacy of BT, suggesting that BT might exert anti-tumor effects mainly by inducing cell apoptosis (Figure 3A).

Hoechst 33258 staining revealed morphological changes, such as cell shrinkage and chromatin condensation, indicating the apoptosis induced by BT (Figure 3B). Additionally, BT treatment also induced apoptosis of U251 cells in a dose-dependent manner (Figure 3C,D). Furthermore, we found that Bcl-2 was significantly decreased, whereas Bax, cytochrome c, cleaved caspase-9, cleaved caspase-3, and cleaved PARP were increased considerably after BT treatment (Figure 3E,F). IHC results showed that Bax and Bcl-2 expressions in tumor tissue significantly increased and decreased, respectively (Figure 3G,H). These results indicated that BT induced apoptosis through the caspase cascade pathway, subsequently leading to the initiation of apoptosis in U251 cells.

### 3.4. BT Inhibits ITGB4/FAK/ERK Pathway

**Figure 4:**
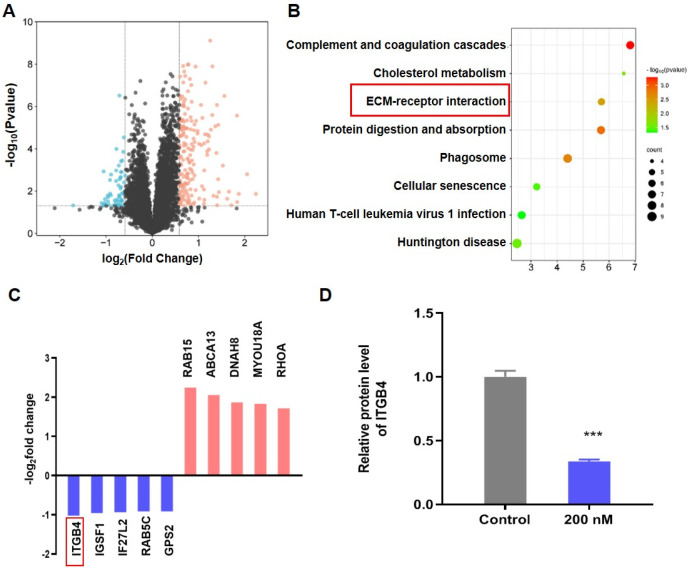
TMT-based quantitative proteomic analysis of BT in U251 cells. (**A**) DEPs were analyzed using a volcano plot. (**B**) KEGG pathway analysis was conducted on DEPs with a red box highlighting the most relevant pathway. (**C**) Top downregulated and upregulated proteins from quantitative proteomic analysis with a red box indicating the most relevant protein. (**D**) Quantitative proteomic analysis of ITGB4 expression. All data were expressed as the mean ± SD of three independent experiments. *** *p* < 0.001 compared with control.

To gain mechanistic insights into BT-induced apoptosis, TMT-based quantitative proteomic analysis was performed to identify key proteins and signaling pathways involved in BT-induced cell apoptosis. 7115 proteins were detected and quantified, of which 176 were significantly upregulated and 57 significantly downregulated (Figure 4A; Tables S1 and S2). KEGG pathway analysis highlighted significant pathways, including ECM-receptor interaction, complement and coagulation cascades, and transforming growth factor-β signaling pathway. The ECM-receptor interaction, which is connected to apoptosis, was the focus of our subsequent investigation (Figure 4B). PPI analysis revealed that ITGB4 was the hub protein in the ECM-receptor interaction pathway (Figure S2). Additionally, ITGB4 was among the top five downregulated proteins (Figure 4C), and quantitative proteome analysis confirmed significant BT-induced downregulation of ITGB4 (Figure 4D).

**Figure 5:**
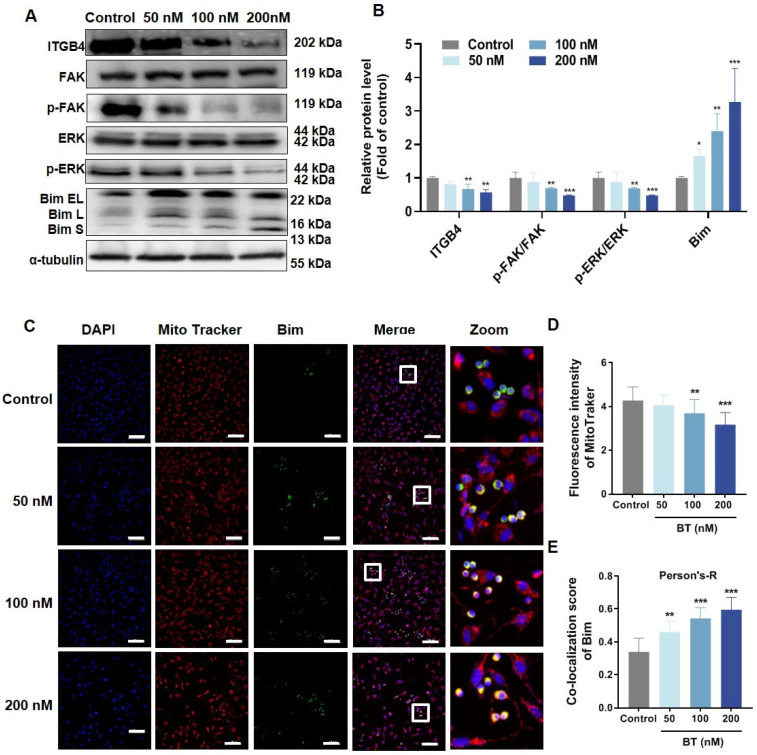
BT inhibits the ITGB4/FAK/ERK signaling pathway. (**A**) Western blot analysis of ITGB4, FAK, p-FAK, ERK1/2, p-ERK1/2, and Bim in U251 cells treated with different concentrations of BT for 48 h. (**B**) Quantitative analysis of the western blot. (**C**) Immunofluorescence analysis showing Bim co-localization in U251 cells (scale bar: 50 μm). (**D**) Quantitative analysis of the relative number of mitochondria with MitoTracker. (**E**) Quantitative analysis of the content of Mito-Bim co-localized in Fig 5C. All data were expressed as the mean ± SD of three independent experiments. * *p* < 0.05, ** *p* < 0.01, *** *p* < 0.001 compared with control.

As an important ECM receptor, ITGB4 plays a crucial role in regulating mitochondrial function and cell apoptosis. Western blotting revealed that BT treatment significantly downregulated ITGB4 and its downstream proteins, including FAK and ERK, whereas the pro-apoptotic Bim significantly increased (Figure 5A,B). In addition, the results of immunofluorescence indicated that BT promoted the nuclear translocation of Bim (Figure 5C,E). Consistently, the relative number of mitochondria characterized by MitoTracker significantly decreased (Figure 5D), indicating that BT exerted anti-tumor effects by destroying U251 cell mitochondria. These results indicated that BT could potentially suppress the expression of ITGB4 and its associated proteins, ultimately initiating apoptosis in U251 cells.

### 3.5. BT Induces Mitochondrial Dysfunction in U251 Cells

**Figure 6:**
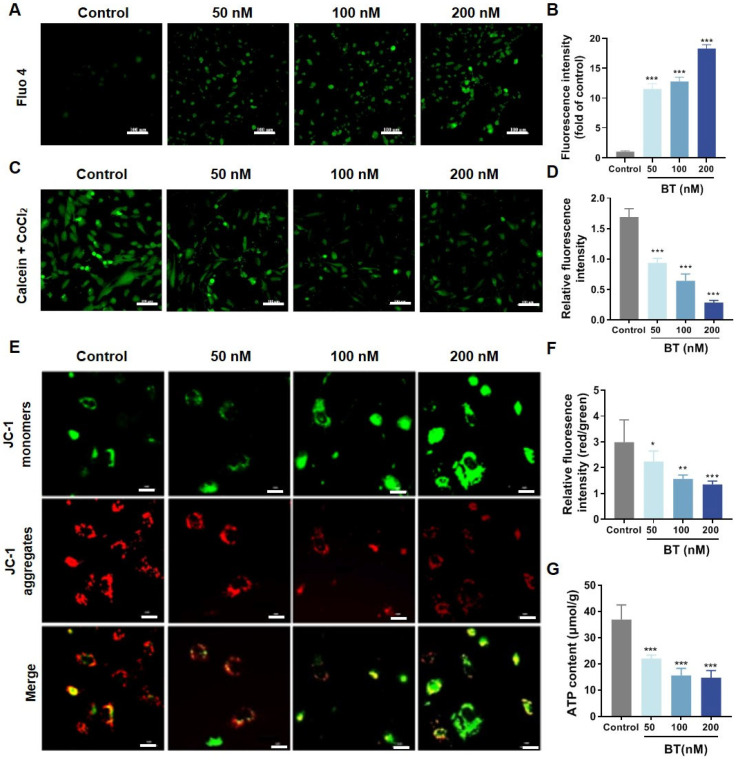
BT-induced mitochondrial dysfunction in U251 cells. (**A**,**B**) Immunofluorescence staining and statistical analysis of Ca^2+^ expression (scale bar: 100 μm). (**C**,**D**) The openness of mPTP was determined using calcein AM/CoCl_2_ fluorescent and statistical analysis of mPTP assay (scale bar: 100 μm). (**E**,**F**) Mitochondrial membrane potential levels were determined using JC-1 fluorescent (scale bar: 50 μm) and statistical analysis of JC-1 assay. (**G**) Intracellular ATP levels in U251 cells. All data were expressed as the mean ± SD of three independent experiments. * *p* < 0.05, ** *p* < 0.01, *** *p* < 0.001 compared with control.

Bim plays a pivotal role in mitochondrial dysfunction [36,37]. Calcium (Ca^2+^) was first detected due to its intricate relation to mitochondrial dysfunction. We observed that intracellular Ca^2+^ levels increased significantly with BT exposure (Figure 6A,B). Next, the opening of the mPTP was examined using calcein AM/CoCl_2_. Under normal conditions, calcein AM is hydrolyzed to calcein, a polar fluorescent dye without membrane permeability that exhibits strong green fluorescence. Once the mPTP opens, CoCl_2_ enters the mitochondria and quenches the green fluorescence induced by calcein. In this study, as shown in Figure 6C,D, the openness of the mPTP significantly increased after BT treatment. Another marker associated with mitochondrial dysfunction, mitochondrial membrane potential (MMP), was examined using JC-1. JC-1 aggregates in the mitochondrial matrix, emitting red fluorescence, whereas when MMP decreases, it disperses into monomers, emitting green fluorescence. As shown in Figure 6E,F, the BT-treated group exhibited stronger green fluorescence and weaker red fluorescence compared to the control group. Quantitative analysis further confirmed that the red/green fluorescence ratio was significantly decreased in the BT-treated group compared to the control group. Besides, BT treatment also significantly downregulated ATP production (Figure 6G). Taken together, the data demonstrated that BT caused mitochondrial dysfunction in U251 cells.

### 3.6. BT Induces Oxidative Stress in U251 Cells

**Figure 7:**
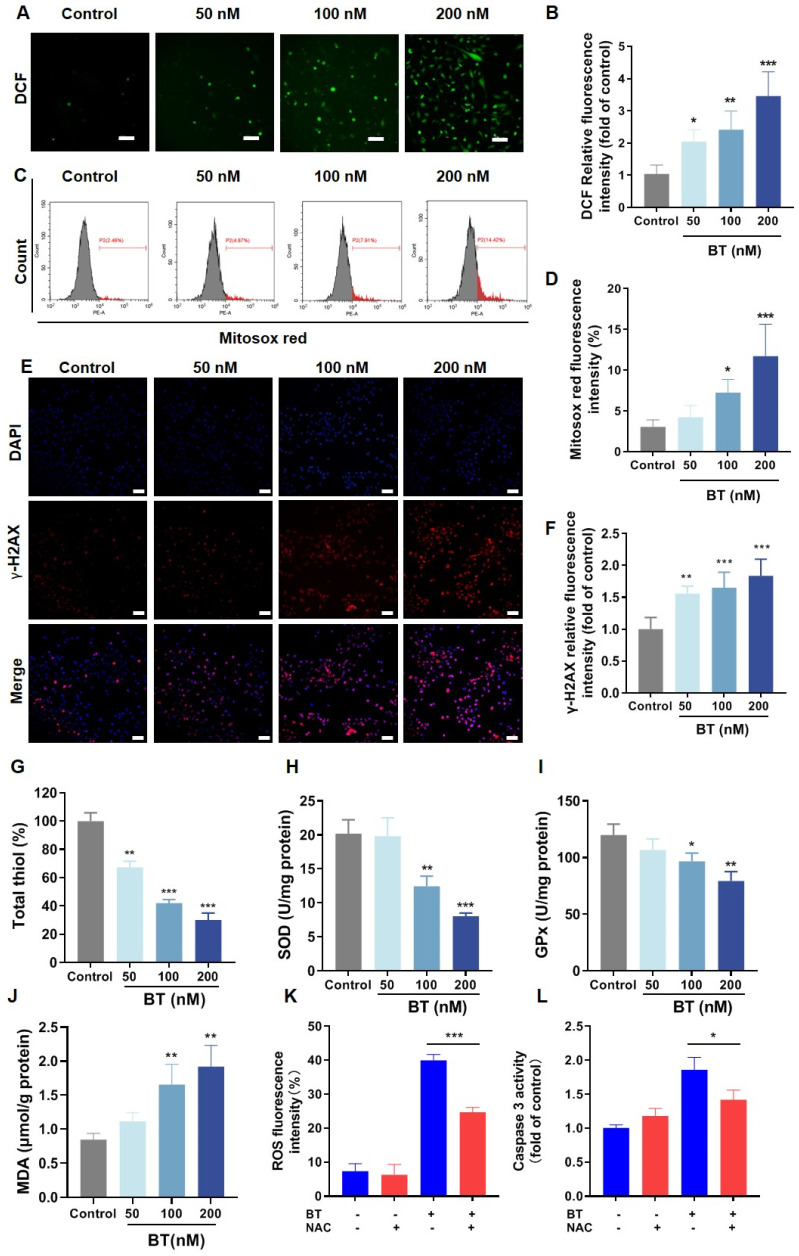
BT induced oxidative stress in U251 cells. (**A**,**B**) Intracellular ROS generation was measured using DCFH-DA. (Scale bar: 100 μm) and quantitative analysis of DCF (Fluorescent substances of DCFH-DA in U251 cells). (**C**,**D**) Superoxide levels in the mitochondria were determined using MitoSox Red. (**E**,**F**) Immunofluorescence analysis of DNA damage biomarker γ-H2AX (scale bar: 100 μm) and statistical analysis of γ-H2AX assay. (**G**) Total thiols in the U251 cells were determined using Ellman’s method. (**H**) Quantification of superoxide dismutase (SOD) activity in U251 cells. (**I**) Quantification of glutathione peroxidase (GPx) activity in U251 cells. (**J**) Lipid peroxidation in U251 cells was determined by the malondialdehyde (MDA) level. (**K**) ROS fluorescence intensity after treatment with a combination of BT (200 nM) and the ROS inhibitor NAC (8 μM) for 48 h. (**L**) Caspase-3 activity after treatment with a combination of BT (200 nM) and the ROS inhibitor NAC (8 μM) for 48 h. All data were expressed as the mean ± SD of three independent experiments. * *p* < 0.05, ** *p* < 0.01, *** *p* < 0.001 compared with control.

Mitochondrial dysfunction is likely to trigger oxidative stress [38,39]. Hence, we examined the ROS level in BT-treated U251 cells by DCFH-DA staining. As shown in Figure 7A,B, the intracellular levels of ROS are significantly increased, which is accompanied by concentration dependence. In addition, we also used the MitoSox probe to detect the ROS level within mitochondria (Mito-ROS), and the results showed that the Mito-ROS also significantly increased (Figure 7C,D), consistent with the ROS level within the whole cell. Next, we tested whether the upregulation of ROS caused by BT treatment could induce DNA damage. γ-H2AX, a phosphorylated form of H2AX, is recognized as a biomarker for DNA damage. Thus, we investigated the expression of γ-H2AX in glioma cells, and the results demonstrated that BT considerably upregulated its expression (Figure 7E,F). In addition, other oxidative stress-related markers, such as total thiol, superoxide dismutase (SOD), glutathione peroxidase (GPx), and malondialdehyde (MDA), were also determined. As shown in Figure 7G–J, BT (100 nM and 200 nM) exposure resulted in a decrease of activities of the vital ROS scavenger (total thiol) and antioxidant enzymes (SOD and GPx), whereas it increased the oxygen radical-induced lipid peroxidation (MDA). To further examine whether the apoptosis induced by BT is associated with ROS, we applied N-acetyl-L-cysteine (NAC), a ROS scavenger, in combination with BT (200 nM) for 48 h. As shown in Figure 7K,L, ROS levels significantly decreased after adding NAC, while caspase-3 activity significantly increased. In conclusion, BT enhances mitochondrial dysfunction-induced oxidative stress in U251 cells.

## 4. Discussion

Despite extensive research, GBM remains a malignant tumor characterized by its invasive nature, with only 6.8% of patients surviving for 5 years following the original diagnosis [40]. Developing novel therapeutic approaches for GBM remains highly challenging. Chansu is widely used in traditional medicine owing to its remarkable therapeutic attributes, including anti-inflammatory, anti-tumor, and antiviral properties [11,41]. As the main component of Chansu, BT holds immense value due to its multifaceted therapeutic potential. For instance, it has been reported that BT-induced non-small cell lung cancer cell ferroptosis significantly inhibited its proliferation by promoting the degradation of GPX4 [16]. In addition, several investigations revealed that BT induced apoptosis in human malignant melanoma A375 cells [17] and multidrug-resistant liver cancer cells [15]. In light of the most recent study, BT promotes mitochondrial dysfunction through the AKT signaling pathway, thereby increasing the chemosensitivity of GBM cells to TMZ [19]. However, they did not further elucidate the causation of mitochondrial dysfunction. In the present study, we systematically explored the potential mechanism, especially the mitochondrial dysfunction and oxidative stress in BT-treated GBM cells.

The extracellular matrix (ECM) is a complex noncellular network composed of glycoproteins, collagens, proteoglycans, and glycosaminoglycans [42]. Various cellular functions, such as survival, adhesion, proliferation, migration, and differentiation, are regulated by the ECM [43]. The ECM-integrin interactions are essential for cell adhesion and regulate actin organization, cell movement, and cell survival [44]. Integrins are imperative cell adhesion receptors that mediate the adhesion and signal transduction of various ECM components [45]. In the current research, we found that ITGB4 is significantly downregulated after BT treatment. As a transmembrane receptor, ITGB4 mainly binds to laminin in the ECM and regulates tumorigenesis and invasiveness of many cancers [46]. Several studies have shown that ITGB4 influences the migration and invasion of tumor cells through the activation of FAK [47,48]. PI3K/AKT and ERK are major downstream signaling pathways that are activated by the FAK. It is known that pro-apoptotic protein Bim is phosphorylated by ERK and degraded by the proteasome [49,50,51]. Consistent with prior findings, our study also showed that BT treatment suppressed the phosphorylation of FAK and ERK, subsequently enhancing the translocation to mitochondria of Bim.

The Bim located at mitochondria directly activates pro-apoptotic proteins Bax and Bak [52], thereby inducing mitochondrial outer membrane permeabilization (MOMP) through their oligomerization. The MOMP leads to the release of intermembrane space proteins, such as cytochrome c, which subsequently activates apoptotic caspases. Besides, excessive mitochondrial Ca^2+^ accumulation triggers mitochondrial permeability transition pore (mPTP) opening, which results in mitochondrial swelling and rupture [53]. In the current work, our data revealed that BT induces mitochondrial dysfunction in GBM cells, as shown by the decrease of mitochondrial membrane potential, Ca^2+^ overload, and mPTP opening. It is widely recognized that mitochondria serve as the primary source of ROS [54]. The transient mPTP opening elicits a rapid ROS release and is promptly scavenged. Conversely, prolonged mPTP opening unleashes a ROS burst, leading to mitochondrial damage. The phenomenon has been termed “ROS-induced ROS release” (RIRR) [55]. Elevating ROS generation plays an essential role in oxidative stress, which can give rise to cellular apoptosis through multiple pathways [56]. In the present work, our results show an elevation in ROS production, accompanied by a decrease in antioxidants such as GPx and SOD, indicating the presence of oxidative stress in BT-treated GBM cells. Recently, a multitude of chemotherapy drugs for GBM have been developed, and a portion of them have been tested in clinical trials. Among these chemotherapy drugs, multiple strategies, such as enzyme and other protein modulators, cytotoxic and cytostatic agents, apoptosis inducers, immunomodulators, and adjuvants, have been employed [57]. Nonetheless, the chemotherapy drugs have not yielded statistically significant advancements in the standard of care for patients with GBM to date. In the current research, our data validate that BT exerts a significant anti-GBM effect by inducing oxidative stress-mediated apoptosis, thereby highlighting BT as a promising therapeutic option for GBM.

## 5. Conclusions

**Figure 8:**
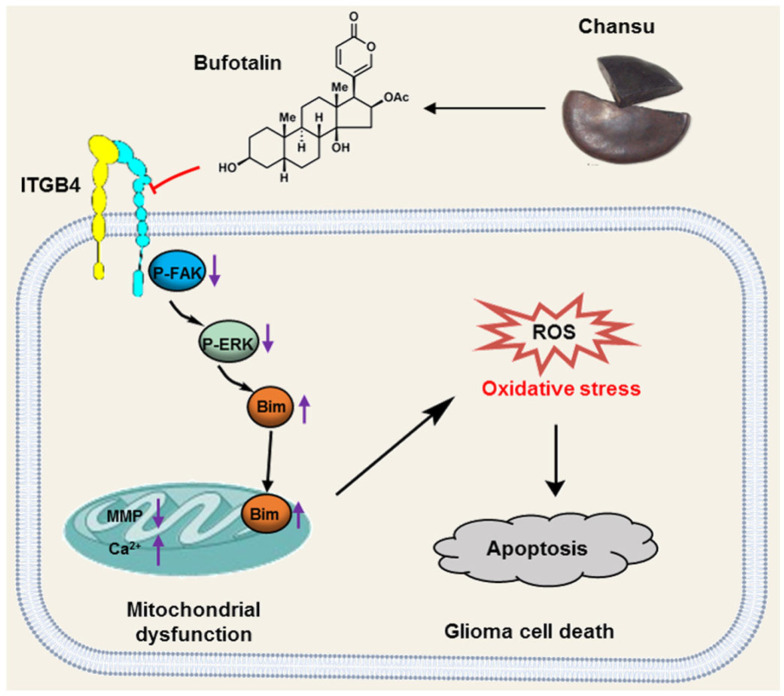
Schematic diagram of the anti-GBM mechanism of BT.

The present study demonstrates that BT effectively suppresses the proliferation of GBM cells and inhibits subcutaneous tumor growth in nude mice. Mechanistically, BT treatment results in mitochondrial dysfunction, triggers excessive production of ROS, and subsequently leads to cell apoptosis. The most notable discovery, however, was the significant downregulation of ITGB4 observed in the context of proteomic-based analysis of differentially expressed proteins following BT treatment. Furthermore, our findings revealed that BT induces oxidative stress-mediated apoptosis by regulating the ITGB4/FAK/ERK signaling pathway (Figure 8). In summary, our study presents compelling evidence for BT’s efficacy in combating GBM, elucidates its underlying mechanism, and underscores BT as a positive lead compound for GBM.

## Data Availability

The data will be made available upon request.

## Supplementary Materials

The following supporting information can be downloaded at: https://www.mdpi.com/article/10.3390/antiox13101179/s1, Figure S1: The cell viability after the combination of BT with ferrostatin-1 or necrostatin-1; Figure S2; PPI analysis of the ECM-receptor interaction pathway; Table S1: Down-regulated proteins in BT-treated U251 cells; Table S2: Up-regulated proteins in BT-treated U251 cells.

## Abbreviations

ATP—Adenosine triphosphate; BT—Bufotalin; CCK-8—Cell counting kit-8; DAB—Diaminobenzidine; DCFH-DA—2′,7′-dichlorofluorescin diacetate; DEPs—Differentially expressed proteins; DMEM—Dulbecco’s modified Eagle’s medium; ECM—Extracellular matrix; ERK—Extracellular signal-regulated kinase; FAK—Focal adhesion kinase; FBS—Fetal bovine serum; H&E—Hematoxylin and eosin; HRP—Horseradish peroxidase; IHC—Immunohistochemistry; ITGB4—Integrin β4; KEGG—Kyoto Encyclopedia of Genes and Genomes; LDH—Lactate dehydrogenase; MDA—Malondialdehyde; MMP—Mitochondrial membrane potential; mPTP—Mitochondrial permeability transition pore; PFA—Paraformaldehyde; PI—Propidium iodine; PPI—Protein-protein interactions; RIPA—Radioimmunoprecipitation assay; ROS—Reactive oxygen species; SOD—Superoxide dismutase; TMT—Tandem mass tag; TMZ—Temozolomide.
