# Supplementary material for: Bufotalin Induces Oxidative Stress-Mediated Apoptosis by Blocking the ITGB4/FAK/ERK Pathway in Glioblastoma
Source: Antioxidants (Basel). 2024 Sep 27;13(10):1179. doi: 10.3390/antiox13101179 (PMC11505062; doi:10.3390/antiox13101179)
Supplement: Supplementary file 1 [file antioxidants-13-01179-s001.zip › antioxidants-3202149-supplementary.pdf]

Bufotalin Induces Oxidative Stress-Mediated Apoptosis by Blocking the ITGB4/FAK/ERK Pathway in Glioblastoma

Junchao Tan, Guoqiang Lin, Rui Zhang, Yuting Wen, Chunying Luo, Ran Wang, Feiyun Wang *, Shoujiao Peng * and Jiange Zhang *

Innovation Research Institute of Traditional Chinese Medicine, Shanghai University of Traditional Chinese Medicine, Shanghai 201203, China; tanjunchao@shutcm.edu.cn (J.T.); lingq@sioc.ac.cn (G.L.); rui_zhang@shutcm.edu.cn (R.Z.); wenyuting@shutcm.edu.cn (Y.W.); 0012021193@shutcm.edu.cn (C.L.); 12022215@shutcm.edu.cn (R.W.)

* **Corresponding authors**

Feiyun Wang: Innovation Research Institute of Traditional Chinese Medicine, Shanghai University of Traditional Chinese Medicine, Shanghai 201203, China. Tel.: +86-021-51323104. E-mail address: yunpumpkin@shutcm.edu.cn

Shoujiao Peng: Innovation Research Institute of Traditional Chinese Medicine, Shanghai University of Traditional Chinese Medicine, Shanghai 201203, China. Tel.: +86-021-51323104. E-mail address: pengshj08@ shutcm.edu.cn

Jiange Zhang: Innovation Research Institute of Traditional Chinese Medicine, Shanghai University of Traditional Chinese Medicine, Shanghai 201203, China. Tel.: +86-021-51323106. E-mail address: jgzhang@shutcm.edu.cn

Contents

Figure S1. The cell viability after the combination of BT with ferrostatin-1 or necrostatin-1.

Figure S2. PPI analysis of the ECM-receptor interaction pathway.

Table S1. Down-regulated proteins in BT-treated U251 cells.

Table S2. Up-regulated proteins in BT-treated U251 cells.

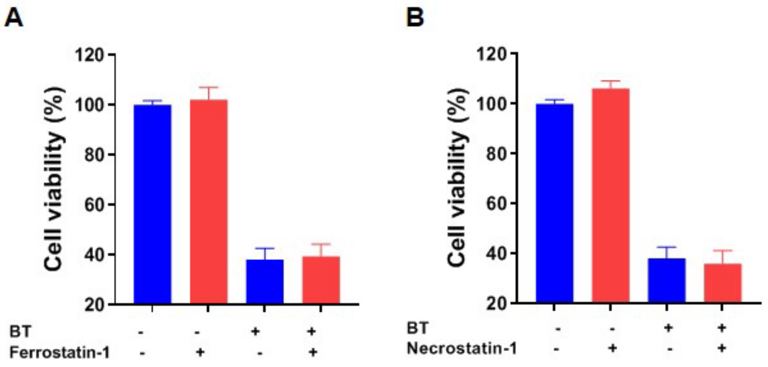

Figure S1. (A) The cell viability after the combination of BT (200 nM) with ferroptosis inhibitor ferrostatin-1 (10 μM) for 48 h. (B) The cell viability after the combination of BT (200 nM) with necrosis inhibitor necrostatin-1 (10 μM) for 48 h. All data were expressed as the mean ± SD of three independent experiments.

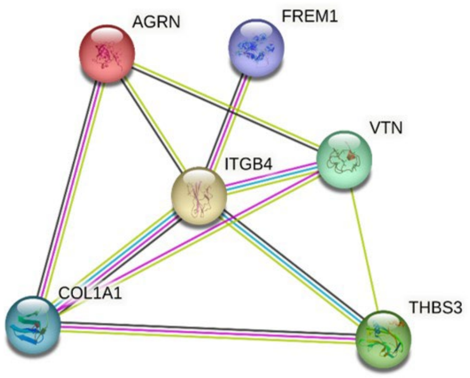

Figure S2. PPI analysis of the ECM-receptor interaction pathway.

Table S1. Down-regulated proteins in BT-treated U251 cells

Number	Protein/Gene	Number	Protein/Gene
1	TKT	30	KCNN4
2	HYOU1	31	BST2
3	ITGB4	32	KDM5A
4	SMARCA4	33	SREK1IP1
5	CPD	34	HLA-C
6	PARP14	35	Q13850
7	GFAP	36	MFAP3
8	IGFBP7	37	COX6C

Number	Protein/Gene	Number	Protein/Gene
9	HIST1H1E	38	B2R6N3
10	A0A142CHG9	39	MANSC1
11	AGRN	40	STK16
12	TXNIP	41	TRPS1
13	COX2	42	IFI27L2
14	COL5A1	43	DGLUCY
15	RAB5C	44	COX18
16	IGFBP2	45	HLA-A
17	MRC2	46	B4DTN1
18	COL11A1	47	SMCO2
19	DTX3L	48	Q2PEG2
20	GNG5	49	GPS2
21	B3KPD8	50	Q3BBV4
22	B2RCN5	51	STON2
23	RPL22L1	52	RBX1
24	EPHA5	53	FAM174A
25	IGSF1	54	ZCRB1
26	CYP27A1	55	SREBF2
27	AKT1S1	56	RIN2
28	SLC1A1	57	TRIM41
29	Q8NAV8		

Table S2. Up-regulated proteins in BT-treated U251 cells

Number	Protein/Gene	Number	Protein/Gene
1	RAB15	89	KIFC1
2	ABCA13	90	C10orf67
3	DNAH8	91	UAP1L1
4	ITGAE	92	CORO1C

Number	Protein/Gene	Number	Protein/Gene
5	MYO18A	93	COL1A1
6	RHOA	94	GBP5
7	AOX1	95	H0YHG0
8	TTN	96	APOE
9	SLC38A2	97	NHEJ1
10	SDHAF3	98	C11orf58
11	AFAP1L1	99	EIF5A2
12	SUI1	100	DENND4B
13	B4DU20	101	NECAP1
14	RPL26L1	102	FMR1
15	CDK15	103	hCG23833
16	MAFF	104	Q53G08
17	SOWAHD	105	FTL
18	CDCA5	106	KCTD4
19	GJA8	107	B3KPQ9
20	MAFK	108	CDCA3
21	B4DH64	109	A8K037
22	HOOK1	110	TUBB1
23	hCG_1991559	111	FEN1
24	HLA-DPB1	112	PLG
25	SAG	113	POLR2D
26	VTN	114	LOC392742
27	TMA7	115	DNMT1
28	B4DDT3	116	B2R5V0
29	APOC3	117	RAD51C
30	SOX3	118	KDELR2
31	C9	119	IFRD1
32	P4HB	120	NFKBIB
33	FAM98C	121	PRC1

Number	Protein/Gene	Number	Protein/Gene
34	NDFIP1	122	C3
35	KIF7	123	B4DXW2
36	LY96	124	BIRC5
37	F5	125	TEX9
38	C9orf72	126	PTTG1IP
39	KIF18B	127	GMNN
40	PCBD2	128	PAQR3
41	T2R1	129	HJURP
42	F10	130	FAM219B
43	B4DJB7	131	UBE2S
44	A4CYL7	132	KIF22
45	SQSTM1	133	PLPP2
46	F13A1	134	HNRPK
47	TARBP1	135	SLC39A8
48	E1A689	136	TMEM127
49	CLIP1	137	LAPTM4A
50	TCF20	138	CDK2
51	CLSPN	139	Q53H66
52	TATDN3	140	SLC6A8
53	GRIA3	141	ANXA5
54	GC20	142	RAB33B
55	APOA1	143	SLC36A1
56	B7Z4Y1	144	C12orf60
57	SHPRH	145	EHD4
58	B3KXW2	146	HCTP4
59	GPX1	147	Q53FB6
60	DIRAS2	148	TNFRSF12A
61	THBS3	149	Q53EZ9
62	WNK2	150	AGFG2

Number	Protein/Gene	Number	Protein/Gene
63	ACT	151	CKAP2
64	ULK1	152	HMCES
65	TAF11	153	PDLIM5
66	ANLN	154	BCL2L12
67	B4DZF2	155	ENC1
68	TMEM59	156	FAM89A
69	N6AMT1	157	B4DWB0
70	TGFB1	158	POTEJ
71	ZNF784	159	LEPROTL1
72	FREM1	160	SMURF2
73	B3KN06	161	B4DXL5
74	PDCD1LG2	162	HLA-C
75	NEBL	163	SDC4
76	DKFZ	164	TMEM237
77	HBA2	165	EIF4E2
78	ZFP42	166	LINGO2
79	PSMA3	167	A8K3S3
80	KIF15	168	BLOC1S2
81	CHRNA9	169	CHEK1
82	TK1	170	IPO5
83	A0A3S8NFS6	171	GEM
84	B3KVU0	172	TAX1BP3
85	NLRP13	173	A8K0I8
86	SFT2D2	174	CWC25
87	ITIH3	175	B4DHK9
88	PPFIA3	176	FTH1
